# Genes, molecules and patients—Emerging topics to guide clinical pain research

**DOI:** 10.1016/j.ejphar.2013.01.069

**Published:** 2013-09-15

**Authors:** Shafaq Sikandar, Ryan Patel, Sital Patel, Sanam Sikander, David L.H. Bennett, Anthony H. Dickenson

**Affiliations:** a Departments of Neuroscience, Physiology and Pharmacology, University College London, WC1E 6BT London, UK; b Nuffield Department of Clinical Neurosciences, West Wing, Level 6, John Radcliffe Hospital, Oxford, OX3 9DU, UK.

## Abstract

This review selectively explores some areas of pain research that, until recently, have been poorly understood. We have chosen four topics that relate to clinical pain and we discuss the underlying mechanisms and related pathophysiologies contributing to these pain states. A key issue in pain medicine involves crucial events and mediators that contribute to normal and abnormal pain signaling, but remain unseen without genetic, biomarker or imaging analysis. Here we consider how the altered genetic make-up of familial pains reveals the human importance of channels discovered by preclinical research, followed by the contribution of receptors as stimulus transducers in cold sensing and cold pain. Finally we review recent data on the neuro-immune interactions in chronic pain and the potential targets for treatment in cancer-induced bone pain.

## 1 Introduction

One of the most important issues in pain research is the translation of basic science findings to the patient, as well as back-translation so that clinical phenomena can be explored and modeled in preclinical studies. Interaction between scientists and clinicians is essential for this process and one obvious shared area of interest is the pharmacological processes that underlie pain conditions. This review selectively explores some areas of pain research that, until recently, have been poorly understood. We have chosen four topics that relate to clinical pain and we discuss the underlying mechanisms and related pathophysiologies contributing to these pain states. A key issue in pain medicine involves crucial events and mediators that contribute to normal and abnormal pain signaling, but remain unseen without genetic, biomarker or imaging analysis. Here we consider how the heritable pain states reveal the importance of channels discovered by preclinical research of pain disorders, followed by the contribution of receptors as stimulus transducers in cold sensing and cold pain. Finally we review recent data on the neuro-immune interactions in chronic pain and the potential targets for treatment in cancer-induced bone pain.

## 2 Familial pain syndromes

Adequate analgesic treatments for a number of chronic pain conditions remain a challenge, partly due to the robust inter-individual variability in sensitivity to pain and analgesics, as well as the individual susceptibility to developing chronic pain Table 1. There is now increasing evidence that a large component of the pain experience is inherited and that pain phenotypes result as a variation in genetic-environmental interactions, including a role for epigenetic factors.

The increasing sophistication and decreasing cost of high-throughput methodologies for identification of genetic components that contribute to human pain disorders have successfully highlighted numerous channelopathies and mutations that underlie familial pain syndromes. Genome-wide linkage mapping, quantitative trait locus mapping and microarray-based gene expression profiling are all advancing techniques, and here we discuss their revelation of some inherited pain states.

### 2.1 Sodium channel Na_v_1.7 mutations

Nine sodium channels have been identified in the nervous system, of which the tetrodotoxin-sensitive Na_v_1.7 channel is expressed in almost all dorsal root ganglia neurones. Na_v_1.7 has fast activation and inactivation kinetics, and is also characterised by slow closed-state inactivation, permitting the channel to respond to small slow depolarisations and thereby acting as a threshold channel to amplify generator potentials to sub-threshold stimuli (Dib-Hajj et al., 2007). Recent human studies have directly linked Na_v_1.7 to four pain disorders: Primary erythromelalgia (PE), paroxysmal extreme pain disorder (PEPD), Na_v_1.7-associated congenital insensitivity to pain (CIP) and small fibre neuropathy (Dib-Hajj et al., 2007; Faber et al., 2012). A difference in perceived pain intensity among neuropathic pain patients is also linked to an *SCN9A* single nucleotide polymorphism and in normal individuals this has been shown to affect heat pain sensitivity (which is predominately C fibre-mediated) (Reimann et al., 2010). PE was the first human pain disorder mapped to an ion channel mutation, where Yang et al. used linkage analysis to identify two missense mutations in the *SCN9A* gene that encodes Na_v_1.7 (Yang et al., 2004).

More than ten independent mutations of *SCN9A* are now linked to PE of varying severity, characterised by intense episodic burning pain and redness in the extremities that are triggered by warm stimuli or exercise (Yang et al., 2004). The clinical onset of PE has been reported in early childhood with severity of pain worsening with age. Effective pain relief can be achieved by repeated immersion of hands and feet in ice-cold water, although this can lead to skin lesions (Michiels et al., 2005). The ‘gain-of-function’ channel mutations underlie hyperexcitability of nociceptors and reduced activation thresholds for action potentials. The redness and swelling of extremities that accompanies PE pain likely involves a dysfunction in sympathetic innervation of the vasculature in affected limbs (Rush et al., 2006).

Another autosomal dominant pain disorder resulting from a different set of ‘gain-of-function’ Na_v_1.7 mutations is PEPD, formerly known as familial rectal pain. PEPD patients suffer from excruciating burning pain and flushing in the anorectal region or around the eyes, also from early childhood (Fertleman et al., 2006). Ocular attacks tend to dominate over rectal pain with increasing age, and these attacks can be triggered by temperature changes and bowel movements.

Functional analysis of mutant channels has revealed a reduction in fast inactivation, giving rise to a persistent current that is not present in the wildtype channel and promotes repetitive firing of nociceptors that underlies paroxysmal pain (Fertleman et al., 2006). Interestingly, most patients with PEPD, but not PE, respond favourably to treatment with carbamazepine due to effective blocking of the persistent current in the PEPD-related M1627K mutant channel (Fertleman et al., 2006).

Estacion et al. (2008) reported a new Na_v_1.7 mutation, A1632E, in a patient with a unique mixture of symptoms that included clinical characteristics of both PE and PEPD. This mutation was localised near the PEPD missense mutation M1627K, resulting in fast inactivation, deactivation, and a persistent inward current. The associated phenotype of A1632E and evidence that alternative splicing can impact the functional consequences of IEM or PEPD mutations suggests that *SC9NA* mutations are likely to produce a broad range of PE- or PEPD-like clinical outcomes with varying severity (Estacion et al., 2008; Jarecki et al., 2009).

A further series of gain of function variants in SCN9a have been associated with the development of small fibre neuropathy. This is a neuropathic pain syndrome associated with distal degeneration of small diameter axons associated with burning pain in the extremities and in some cases autonomic dysfunction. These variants produce gain of function changes rendering dorsal root ganglion neurons hyper-excitable (Faber et al., 2012). In some cases variants render superior cervical ganglion neurons hypoexcitable and these are associated with more severe autonomic symptoms (Han et al., 2012).

CIP is associated with ‘loss-of-function’ mutations of Na_v_1.7 that produce a profound insensitivity to pain from birth, whilst retaining intact functionality of all other sensory modalities (Cox et al., 2006). Affected individuals have been reported to display painless burns and fractures with no response to injury or noxious stimulation, but still appear to exhibit normal autonomic and motor responses. Cox et al. studied three families and identified three homozygous nonsense mutations of Na_v_1.7 – S459X, I767X, and W897X – that produced truncated, non-functional proteins, and a further nine have been reported (Cox et al., 2006; Goldberg et al., 2007). Preclinical studies using Na_v_1.7 nociceptor-specific knockouts reproduce a pain insensitive phenotype, thus validating the crucial requirement of Na_v_1.7 function for nociception (Nassar et al., 2004).

The spectrum of clinical phenotypes of Na_v_1.7 channelopathies remains intriguing, ranging from gain-of-function mutations to produce localised pain (IE and PEPD) to loss-of-function mutations resulting in a global insensitivity to all modalities of pain. Mutations of *SCN9A* have also been linked to febrile seizures (Singh et al., 2009), and so the extensive effects *SCN9A* channel mutations on neuronal excitability implicate essential roles for Na_v_1.7 function in human neurophysiology, and distinctly, nociceptive neurotransmission.

### 2.2 Calcium channels and inherited migraine

As with sodium channels, mutations of voltage-gated calcium channels also underlie several inherited diseases, including migraine, cardiac arrhythmia and periodic paralysis. Sodium and calcium channels share some structural similarities; the pore-forming α1 subunits of voltage-gated calcium channels resemble the α subunits of sodium channels with four internally repeated domains (I–IV) with associated auxiliary units (Pietrobon, 2005). The Ca_v_2 subfamily (Ca_V_2.1, Ca_V_2.2, and Ca_V_2.3) comprises the primary calcium channels that initiate neurotransmitter release at fast conventional synapses, and mutations of Ca_v_2.1, located in somatodendritic membranes and in high density in presynaptic terminals throughout the CNS, have been implicated in inherited migraine (Pietrobon, 2005).

Migraines affect more than 10% of the population and research implicates the cause of these disabling, episodic headache pains to activation of the trigeminovascular system and meningeal nociceptors, as well as sensitisation of medullary dorsal horn neurones (Olesen et al., 2009). Familial hemiplegic migraine (FHM) is a rare autosomal dominant form of migraine that is associated with moderate to severe hemiplegia, ataxia and seizures.

FHM is linked to three genes; the first, *CACNL1A4* (FHM1) codes for the α1 subunit of the Ca_v_2.1 neuronal voltage-gated calcium channels (Ophoff et al., 1996). The majority of the 25 known mutations result in a gain-of-function phenotype, where the activation threshold for the channel is reduced (De Vries et al., 2009). Knock-in mouse models of the disease carrying the human FHM1 R192Q or S218L mutations show reduced threshold and increased strength of excitatory neurotransmission in thalamocortical neurones that could mediate cortical spreading depression, the phenomenon that underlies migraine aura (Tottene et al., 2009; van den Maagdenberg et al., 2004). In individuals with visual disturbances, cortical spreading depression originates as a short burst of depolarisations from the occipital lobe, self-propagating towards the frontal cortex (van den Maagdenberg et al., 2004). Other genetic factors relating to migraine are associated with missense mutations of *ATP1A2* (the Na^+^/K^+^ ATPase ion channel pump α2 subunit; FHM2) and *SCN1A* (Na_v_1.1 sodium channel; FHM3) (Dichgans et al., 2005; Segall et al., 2005).

The facilitations of cortical spreading depression in FHM1 mice link increased cortical excitation to abnormal sensory processing in migraine. Changes in synaptic strength and neuronal excitability that produce cortical hyperexcitability and CSD are likely to be key targets for novel preventive migraine treatment (Tottene et al., 2009).

### 2.3 Hereditary and sensory autonomic neuropathy

The hereditary sensory and autonomic neuropathies (HSANs) comprise a group of clinically heterogeneous disorders associated with sensory dysfunction and varying degrees of autonomic dysfunction. Loss of sensation, lancinating pain (especially related to SPTLC1 mutations) and autonomic dysfunction are the most common symptoms of HSANs. Associated skin injuries can lead to chronic skin ulcers, osteomyelitis and extrusion of bone fragments. The most common, HSAN-1 (hereditary sensory radicular neuropathy), involves progressive degeneration of dorsal root ganglion and motor neurones, leading to distal sensory loss and later distal muscle wasting and weakness (Nicholson et al., 1996). The autonomic effects are variable across HSAN types, but can include altered hyperhydrosis/anhydrosis, cardiovascular dysregulation and gastrointestinal dysmotility.

Patients with autosomal dominant forms of HSAN typically show juvenile- or adult-onset sensory neuropathy. By contrast, the autosomal recessive forms of HSAN typically show an earlier onset (Rotthier et al., 2012). Positional cloning and functional candidate-gene approaches have led to the identification of 12 causal genes for HSANs; one interesting mutation is of nerve growth factor (NGF) gene *NGFB*, underlying the development of HSAN-4 (Carvalho et al., 2011). NGF belongs to the neurotrophin family of proteins that regulate neuronal survival, development and function. In adults, NGF is a potent mediator of pain; it mediates inflammatory and immune responses following tissue injury by initiating and maintaining peripheral sensitisation (Nicol and Vasko, 2007). It is presumed the HSAN phenotypes are related to binding of mutant forms of NGF to its receptors.

NGF signals through both the TrkA receptor and the low-affinity NGF receptor p75^NTR^, which facilitates interactions between β-NGF and Trk-A and also enables Trk-A-independent signalling (Nicol and Vasko, 2007). The p75^NTR^ signaling pathway leads to an increase in ceramide and sphingosine-1-phosphate levels, which evokes sensitisation of peripheral neurones. In vitro studies have shown lower binding affinities of the mutant β-NGF to p75^NTR^ and its failure to evoke nociception when injected into mice (Capsoni et al., 2011; Covaceuszach et al., 2010). Moreover, β-NGF secretion is more pronounced in the frameshift mutation compared to missense mutants, which is likely related to the more severe phenotype of patients with frameshift mutations compared to patients with missense mutations (Carvalho et al., 2011). Better understanding of NGF interactions in the nervous system will clarify how its reduced efficiency in HSANs contribute to neuropathic effects.

### 2.4 Familial episodic pain syndrome

Transient receptor potential (TRP) channels are cation channels that have multiple roles in sensory transduction, including mechanosensation, thermosensation, vision, olfaction and chemosensation (Nilius, 2007). TRP mutations have been associated with various human physiological disorders, i.e. mutations in TRPV4 are linked to two neurodegenerative diseases, scapuloperoneal spinal muscular atrophy and Charcot–Marie–Tooth disease type 2C (Deng et al., 2010; Landoure et al., 2010), as well as skeletal dysplasias (Krakow et al., 2009). Yet only one TRP mutation has been associated with a pain syndrome that was first identified in a Colombian family with familial episodic pain syndrome (FEPS) (Kremeyer et al., 2010). FEPS involves painful episodes triggered by conditions of fatigue, fasting, and cold, resulting in severe pain localised principally to the upper body. It is associated with a missense gain-of-function mutation in the *TRPA1* gene (N855S), attributing a five-fold increase in activation current of the channel (by cold or chemical stimuli) at normal resting potential. TRPA1 is expressed in primary afferent nociceptors in rodents and man, and excessive activity in these sensory afferents is thought to underlie the spontaneous pain episodes experienced by FEPS patients, although the precise mechanisms linking the channelopathy with FEPS remain unclear. The rising popularity of genome-wide association studies is likely to illuminate how other TRP channel mutations also contribute to human pain states.

### 2.5 Discussion

Although the genetic components underlying some familial pain syndromes have been identified, the alterations in nociceptor excitability at the molecular and systems levels underlying pathophysiology are not well understood. SCN9A provides a fascinating example whereby distinct mutations can produce episodic pain conditions with very different anatomical distributions or even a distal degeneration of small diameter axons. Nevertheless, a key element of the inherited pain states reviewed here is the imbalance between excitability and inhibition of nociceptive pathways. Some studies are still at an early stage, but the advancement of genetic profiling techniques will yield key insights that lead to deeper mechanistic understanding of the pathophysiology underlying these familial pains, and eventually lead to novel and effective analgesic therapies.

## 3 Molecular and neuronal components of cold sensory processing

Sensory afferents can detect a wide range of temperature changes—a process shown to be predominantly dependent on transient receptor potential channels. Cold temperatures can elicit a range of sensations from pleasant, refreshing and cooling to aching, pricking and somewhat paradoxically, a sensation of burning. Abnormal cold sensitivity is a common feature of chemotherapy-induced neuropathy, although indeed, treatment is often restricted owing to neurotoxic side effects. Estimates suggest a 19% prevalence of cold allodynia among patients with neuropathy (Maier et al., 2010), yet very little is known of the mechanisms involved in its manifestation or cold detection compared to the transduction of heat stimuli.

### 3.1 Primary afferent fibres and central pathways

Defining the boundaries between innocuous to noxious cold is complex as cold pain thresholds can vary according to the rate of cooling (Harrison and Davis, 1999). Innocuous cool is commonly defined as temperatures between 30 °C and 15 °C, whereas noxious cold is generally perceived at temperatures below 15 °C. In primates, cold responsive fibres have been identified with receptive fields consisting of one or many cold spots (Kenshalo and Duclaux, 1977) and are thought to conduct in the Aδ and C fibre ranges. In the rat, a subset of slowly adapting mechanosensitive Aδ fibres can be excited by noxious cold, and in humans these may be responsible for pricking sensations given the reduction of cold sensation following A fibre block (Simone and Kajander, 1997). Microneurography has been used to isolate C fibres in human skin responding to innocuous and noxious cold stimulation (Campero et al., 2001, 1996). Although low threshold C fibres are excited by cooling, the perception of innocuous cooling is likely to be dependent on Aδ fibres; selective A fibre block from pressure and ischemia results in a loss of touch and cool sensitivity and a perception of burning in response to cold stimulation (Simone and Kajander, 1997). This is likely mediated by a release of central inhibition of C fibres by A fibres, thereby unmasking a burning pain sensation (Yarnitsky and Ochoa, 1990). This can be experienced with low concentrations of topically applied menthol, whereas higher concentrations can produce cold and mechanical allodynia in control uninjured areas and paradoxical analgesia in injured areas in neuropathic subjects (Wasner et al., 2008).

Lamina I neurones in the superficial dorsal horn receive convergent input from Aδ and C fibres, and cold stimuli can induce Fos expression in lamina I neurokinin 1 receptor-expressing neurones that is graded with stimulus intensity (Doyle and Hunt, 1999). Furthermore, exposure of rats to ambient cool temperatures induces Fos expression in parabrachical and hypothalamic neurones—also the projection targets of lamina I neurones (Kiyohara et al., 1995). Electrophysiological studies indicate that 86% of wide dynamic range neurones in the rat deep dorsal horn respond to cold and heat stimuli in an intensity-dependent manner (Khasabov et al., 2001). In humans, functional magnetic resonance imaging of the brain reveals common areas of activation following noxious heat (46 °C) and noxious cold (5 °C) stimulation, including the thalamus, insula, and cingulate, somatosensory, premotor and motor corticies (Tracey et al., 2000). Spinal processing of sensory information is under dynamic descending modulation by supraspinal structures, and therefore stimulation of the periaqueductal gray can selectively inhibit spinal neurones responding to noxious cold stimulation (Leith et al., 2010). Lidocaine block of the rostral ventromedial medulla has also been shown to attenuate cold hypersensitivity in models of neuropathy (Taylor et al., 2007).

### 3.2 Cold transduction and hypersensitivity

At the molecular level, cold is detected by the transient receptor potential melastatin 8 (TRPM8). This non-selective cation channel is activated by menthol, is expressed on a subset of nociceptive afferents and has an activation threshold below 20 °C (McKemy et al., 2002; Peier et al., 2002). TRPM8 is a six transmembrane channel with C and N terminals located intracellularly and is thought to form functional channels as tetramers (Stewart et al., 2010). Temperature-dependent gating is conferred by structures contained in the C terminus as demonstrated by chimeras of TRPM8 and TRVP1 channels (Brauchi et al., 2006). TRPM8 channels can undergo post-translational modification; *N*-glycosylation facilitates trafficking of channels to the membrane (Erler et al., 2006), whereas removal of the N934 *N*-glycosylation site results in a shift in the voltage dependency and decreased responses to cold and menthol (Pertusa et al., 2012). TRPM8 is also subject to intracellular modulation by phosphatidylinositol 4,5-bisphosphate (PIP_2_) and phospholipase C, which underlie calcium-mediated adaption to cold mimetic compounds such as menthol (Daniels et al., 2009).

Consistent with the expression of TRPM8 in nociceptive neurones, TRPM8-deficient mice have dramatically reduced cold-sensitive dorsal root ganglia neurones, and show severe deficits in behavioral cool thermosensation (both acute and in response to injury) (Bautista et al., 2006; Colburn et al., 2007; Dhaka et al., 2007). Moreover, behavioural tests in TRPM8 and TRPA1 double knockout mice suggest that aversion to noxious cold is dependent on TRPM8 and not TRPA1 (Knowlton et al., 2010), although menthol has been shown to reversibly block TRPA1 in rodents (Karashima et al., 2007).

Initially, immunohistochemical analysis of dorsal root ganglia in the mouse revealed expression of TRPM8 in a subpopulation of primary afferents distinct from neurones expressing the heat sensor VR1, as well as the nociceptive markers CGRP and IB4 (Peier et al., 2002). TRPM8 protein and mRNA has also been detected in rat arterial myocytes, implicating TRPM8 in the regulation of vasomotor responses to cooling (Johnson et al., 2009). On the other hand, cells counts of menthol- and capsaicin-responsive dorsal root ganglia nociceptive neurones in culture have reported 50% of TRPM8-expressing neurones also expressing vanilloid receptor 1 (TRPV1) (Babes et al., 2004; McKemy et al., 2002). Moreover, the peripheral and central projections of TRPM8 positive neurones have been identified with the insertion of GFP at the TRPM8 locus (Dhaka et al., 2008). TRPM8 afferent terminals target the superficial layer of the epidermis, including mystacial pads, and TRPM8-expressing neurones also predominantly project to lamina I in the dorsal horn. This study also confirmed previous findings that TRPM8 positive neurones are not CGRP, IB4 or NF150 positive, although did report co-expression of TRPM8 and TRPV1 that increases following inflammatory insult (Peier et al., 2002; Story et al., 2003). This co-expression may underlie the paradoxical burning sensation associated with noxious cold, however the perceptual outcomes relating to the quality of a sensation are confounded by relative contributions of sensory coding in the periphery versus thalamocortical processing.

Indeed, the use of cold mimetic compounds is now validated as an effective tool to induce and investigate cold hypersensitivity. A high concentration of 40% topical l-menthol application has been used in humans to produce cold hyperalgesia, increased mechanical sensitivity and pinprick hyperalgesia, substantiating a role for both sensitisation of C fibres and activation of Aδ fibres in cool sensory processing (Binder et al., 2011; Wasner et al., 2008).

### 3.3 Cold and analgesia

Although cold stimulation can be nociceptive and produce hypersensitivities, it is well known that cooling compounds can also produce pleasant sensations and even analgesia. Over the counter topical applications of menthol in the form of creams and patches are readily available to provide pain relief. Some clinical trials employing topical menthol administration include patients with neck pain (3.5% topical menthol to the upper trapezius and neck muscles; ClinicalTrials.gov identifier: NCT01542827) and patients with migraine without aura (10% menthol applied to the forehead and temporal area; (Borhani Haghighi et al., 2010). Topical menthol has also been reported to provide pain relief in chemotherapy-induced peripheral neuropathy following application to areas of pain or sensory disturbance (Storey et al., 2010).

Electrophysiological characterisation of rat spinal neurones and behavioural tests reveal a biphasic effect of topical menthol on cold-evoked responses, reducing the thermal thresholds and avoidance of colder temperatures at low concentrations, whereas increased thermal thresholds and enhanced cold avoidance are reported at higher concentrations (Klein et al., 2010, 2012). It is possible that the inhibitory effects of menthol at higher concentrations are related to penetration of the skin to intradermal nerve endings and the recruitment of more primary afferents, and given the co-expression of TRPA1 and TRPV1 (Story et al., 2003, Salas et al., 2009), menthol may indirectly affect TRPV1 via inhibition of TRPA1. Nonetheless this does not correlate with human pain threshold with respect to the nociceptive effects of high topical menthol concentrations, as well as the lack of reduction in TRPA1 activity with increasing concentrations of menthol (Binder et al., 2011; Xiao et al., 2008). Instead, GABAergic inhibition of peripheral nociceptors may be enhanced (Hall et al., 2004). Moroever, inhibitory neurones in the superficial dorsal horn may recruited proportionally with increasing concentrations of peripherally applied menthol to inhibit cold-evoked firing of dorsal horn neurones (Takazawa and MacDermott, 2010).

Following sciatic nerve ligation, intrathecal or peripheral administration of the cold mimetic compounds menthol and icilin can suppress thermal and mechanical hypersensitivity, an effect reversed by intrathecal antisense knockdown of TRPM8 (Proudfoot et al., 2006). The analgesic effect of TRPM8 activation is centrally mediated and is thought to rely on type II/III metabotropic glutamate receptors and not endogenous opioid signaling, given the failure of naloxone to reverse the analgesic behavioural effects of cooling compounds. While cooling is analgesic in both phases of the formalin test in wildtype mice, deletion of TRPM8 inhibits cooling-induced analgesia in the first but not second phase, suggesting a key role of TRPM8 afferents in the induction of cold-sensing (Dhaka et al., 2007).

Activation of TRPM8 by menthol shifts the channel voltage dependence to negative potentials, thereby increasing channel opening at physiological temperatures. Antagonising channel activity, i.e. with PBMC, shifts voltage dependence towards more positive potentials and so PBMC treatment produces a dose-dependent hypothermia in wildtype animals while TRPM8-knockout mice remained unaffected (Knowlton et al., 2011). Systemic PBMC also diminished cold hypersensitivity in inflammatory and nerve-injury pain models. However, studies using various TRPM8 modulators are often complicated by actions at other TRP channels despite low homology between channels, rendering the selective functional analysis of the channel difficult. Nevertheless like TRPV1, TRPM8 is necessary for thermoregulation, and the inhibition of peripheral but not central channels is required for the hypothermic effects of TRPM8 antagonists (Almeida et al., 2012).

Among the voltage-gated sodium channels, Na_v_1.8 is expressed exclusively in a subset of small nociceptive afferents (Djouhri et al., 2003) and Na_v_1.8 knockout mice are almost completely unresponsive to noxious cold as shown by the cold plate test and also have impaired responses to noxious mechanical stimuli (Zimmermann et al., 2007). Unlike other sodium channels, the inactivation kinetics of Na_v_1.8 are resistant to cold, which indicates a critical role Na_v_1.8-expressing neurones for the detection of noxious cold. Furthermore, menthol is a state-selective blocker of the tetrodotoxin-resistant Na_v_1.8 and Na_v_1.9 sodium channels, further indicating a role for sodium channel blockade in the efficacy of menthol as topical analgesic compound (Gaudioso et al., 2012).

### 3.4 Other leading candidates for cold transduction

A significant proportion of rodent DRG neurones are excited by cooling but are insensitive to menthol (Munns et al., 2007). It is clear that other transducers of noxious cold exist, though these are yet to be identified.

Transient receptor potential subfamily A member 1, TRPA1, is another TRP cation channel with an activation threshold below 17 °C and is expressed in peptidergic nociceptors expressing TRPV1 (Story et al., 2003). It is targeted by pungent irritants from mustard and garlic to produce inflammatory pain, and in human tissue, TRPA1 activation by intracellular Ca^2+^ can occur via an EF-hand domain to produce cold sensitivity (Bautista et al., 2006; Zurborg et al., 2007). It is thought that TRPA1 activation maybe increase peripheral drive in addition to primary afferent activity linked to TRPM8 activation, yet the potential role of TRPA1 as a sensor of noxious cold is controversial (Bautista et al., 2006; Kwan et al., 2006; Nagata et al., 2005). Nevertheless, cold plate and tail-flick experiments reveal TRPA1-dependent, cold-induced nociceptive behaviour in mice, and a subset of cold-sensitive trigeminal ganglion neurones are reported absent in TRPA1-deficient mice (Karashima et al., 2009).

TRPC5 channels were discovered to be gated by cooling in the range of 37–25 °C (Zimmermann et al., 2011). Although TRPC5 knockout mice have no overall changes in thermal and mechanical thresholds, peripheral nerve recordings reveal that TRPM8-expressing afferents form a larger component of cold sensing.

Inhibition of K^+^ leak channels has been proposed to be involved in cold transduction. Differences in potassium currents identified between cold sensitive and cold insensitive trigeminal neurones suggest the presence of a 4-AP-sensitive potassium current in cold insensitive neurones acting as an ‘excitatory brake’ to prevent excitation during cooling (Viana et al., 2002). Cold hypersensitivity may result as a loss of this ‘brake’ in high threshold receptors to produce cold allodynia, i.e. in neuropathic pain. The 2 pore domain potassium channels TREK and TRAAK are expressed in sub-populations of TRPV1-, TRPV2- and TRPM8-expressing rat trigeminal neurones. Both potassium channels have been implicated in modulating mechanical, heat and cold nociception (Alloui et al., 2006; Maingret et al., 1999). TREK1/TRAKK double knockout mice exhibit increased thermal hyperalgesia, increased cold avoidance and cold hypersensitivity after nerve injury, suggesting TREK-1 and TRAAK may in tandem modulate cold transmission (Noël et al., 2009).

### 3.5 Discussion

A body of evidence links TRPM8 to the core of cold sensory processing from innocuous cooling, pain and analgesia. Background potassium currents, intracellular modulation and modification of channels and differential expression on subgroups of primary afferent fibres could in part explain how one molecular entity can confer multiple aspects of cold sensing. Given the specialised nature of cold fibres in the periphery and the emergence of more selective modulators of channel activity, TRPM8 has become an attractive target for the treatment of abnormal cold sensitivity.

## 4 Immune cells and their interactions with nociceptive signaling

Immune cells can influence neuronal function in various pain states. Indeed, the activation of inflammatory cells is classically associated with pain with respect to heat, swelling and abnormal sensations. More recent research implicates immune cell activity not only in inflamed tissues, but also in damaged peripheral nerves and in the central nervous system. Here we review a host of immune cells that are recruited during the inflammatory response after tissue or nerve injury, followed by the release of numerous chemical messengers that contribute to inflammation and activation of associated nociceptive pathways.

### 4.1 Neutrophils

**Fig. 1:**
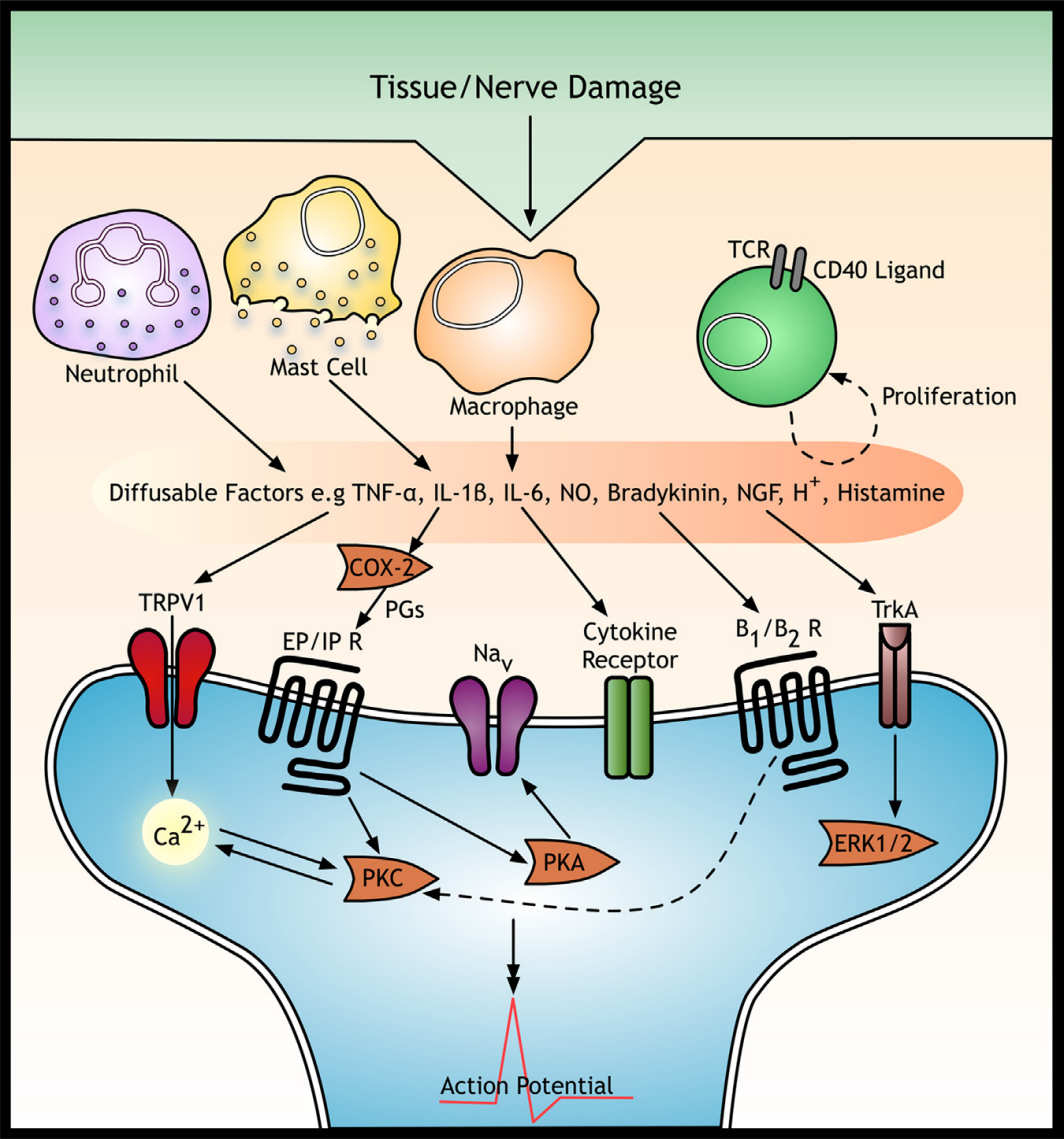
Neuro-immune interactions in the periphery. Resident immune cells are present in the skin, nerve and DRG, surveying tissue for damage or disease. After tissue or nerve injury, these cells release inflammatory mediators and further immune cells are recruited from the vasculature. Lymphocytes are recruited later and proliferate to amplify the immune response. Collectively these cells release cytokines and chemokines resulting in increased expression of neuronal receptors for these inflammatory mediators and increased postsynaptic excitability of sensory neurones. TNFα: tumour necrosis factor-α; IL-1β: interleukin-1β; IL-6: interleukin-6; NO: nitric oxide; COX2: cyclooxygenase 2; TRPV1, transient receptor potential channel; B1/B2: bradykinin receptor; EP/IP: prostanoid receptor; ERK1/2: extracellular signal-regulated kinase 1/2; Na_v_: voltage-activated sodium channel; PGs: prostaglandins; PKA/PKC: protein kinase A/C; TrkA, tyrosine receptor kinase A. (Adapted from Marchand et al. (2005))

Neutrophils (or polymorphonuclear leukocytes) are the earliest inflammatory cell to infiltrate tissue and dominate the acute inflammatory stage to play an important role in early phagocytosis (Ainsworth et al., 1996). Neutrophils also release various inflammatory mediators and chemotactic factors, including lipoxygenase products, nitric oxide, cytokines (e.g. interleukin-1 (IL-1) and tumour necrosis factor-α (TNFα)), chemokines (e.g. IL-8) and growth factors (G-CSF and GM-CSF). They are essential for recruitment of other immune cells and triggering the onset and amplification the inflammatory response (Fig. 1).

Neutrophils use selectins and B2 integrins to extravasate from the blood in order to seek and neutralise targets. The adherence of neutrophils is a highly regulated process initiated by ‘rolling’ along the luminal surfaces of capillaries which allows these leukocytes to probe the endothelium and survey the environment, permitting immediate responses to inflammation via transendothelial migration (Kishimoto et al., 1991). Released cytokines not only enable adherence to endothelial cells with the production of reactive oxygen species, but also attract other inflammatory cell types, including macrophages, to mediate inflammatory hypersensitivity (Witko-Sarsat et al., 2000).

In rodent models of inflammatory pain that are induced with the injection of antigens, including zymosan, lipopolysaccharide (LPS) or carageenan, the accumulation of neutrophils in the treated tissue is critical for the development of evoked hypersensitivity and standard inflammatory markers (Cunha et al., 2008). In the intact uninjured nerve there in an absence of neutrophils, however in rodent models of neuropathy (including partial nerve ligation (Zuo et al., 2003), sciatic nerve crush (Perry et al., 1987) and chronic constriction injury (Clatworthy et al., 1995)), neutrophils migrate and infiltrate the site of the nerve lesion. Endoneurial neutrophil invasion is thought to play a critical role in the development of guarding behavior and thermal hypersensitivity (Perkins and Tracey, 2000). Cumulatively the preclinical data suggests that neutrophils may be important during the early stages of neuropathic pain development, and their release of chemokines at the injury site initiates a later accumulation of macrophages and T-cells (Scapini et al., 2000).

### 4.2 Mast cells

Mast cells are critical resident effector cells for the allergic response and are crucial for innate immunity (Galli et al., 2005). In mammals, mast cells are widely distributed throughout vascularised tissues and peripheral nerves (Galli et al., 2005). Adenosine (Sawynok et al., 2000) and bradykinin (McLean et al., 2000) are likely activators of mast cells following nerve injury to induce degranulation and associated release of pro-inflammatory and nociceptive mediators such as histamine, serotonin, nerve growth factor (NGF) and cytokines (Galli et al., 2005; Metcalfe et al., 1997). Some of these inflammatory mediators are stored in cytoplasmic granules, whereas others, like cytokines, exist as precursors in the cell or are attached to the cell (Stassen et al., 2002). Mast cell degranulation is associated with vascular changes and recruitment of other immune cell types including neutrophils and macrophages to amplify the inflammatory response and activity within nociceptive circuits (Perkins and Tracey, 2000).

Activation of mast cells in human skin with compound 48/80 (a polyamine that causes degranulation) produces erythema, profound itch and marked thermal hyperalgesia (Drummond, 2004). Further evidence for a role of mast cells in nociceptive processing has been implicated among patients with interstitial cystitis and chronic pancreatitis, who show a 3.5-fold increase in the number of mast cells compared with patients without pain (Hoogerwerf et al., 2005; Oberpenning et al., 2002).

In the golgi apparatus of mast cells, histidine is decarboxylated to form histamine (White et al., 1987) that upon cutaneous application to human skin produces a wheal, flare and distinct pruritic sensations (Sikand et al., 2011). In neuropathic pain patients, the processing of pruritic sensation is significantly altered and cutaneous histamine results in a severe increase in spontaneous burning pain (Baron et al., 2001). In a rodent model of peripheral nerve damage using a crush injury, an upregulation of H_1_ receptors in small DRG neurones heightens sensitivity and evoked activity of sensory neurones to histamine (Kashiba et al., 1999).

The histamine H₄ receptor mediates several histamine-induced cellular functions of leukocytes, including cell migration and cytokine production, yet histamine signaling through the H₄ receptor can also have anti-pruritic and anti-nociceptive functions as revealed by the H₄ antagonist INCB38579 that can reduce histamine-induced itch in mice and carrageenan-induced acute inflammatory pain in rats (Shin et al., 2012).

Mast cell-deficient mice are unable to develop the appropriate pain behaviour and pathophysiology following of interstitial cystitis as well as the appropriate thermoregulatory responses during sepsis (Nautiyal et al., 2009; Rudick et al., 2008). This body of evidence supports the role of mast cells in triggering the inflammatory response and pursuing nociceptive activity.

### 4.3 Macrophages

Under normal physiological conditions, macrophages are responsible for interstitial homeostasis by removing cellular debris. In primed immune states, resident and blood-recruited macrophages phagocytose foreign material, microbes, other leukocytes and injured tissue i.e., during Wallerian degeneration (Bruck, 1997). Endogenous (i.e., pro-inflammatory factors released by necrotic cells) and exogenous signals (foreign agents) can activate macrophages, followed by their migration to the site of injury to release pro-inflammatory mediators (cytokines TNFα and IL-1β, NGF, nitric oxide and prostanoids).

The accumulation of macrophages and the release of mediators have been shown to modulate pain processing experimentally. The overt pain behaviour induced by intraperitoneal injections of acetic acid, LPS or zymosan in rodents is exacerbated with increasing macrophage concentrations, partly attributed to the release of TNFα and IL-1β (Ribeiro et al., 2000; Thomazzi et al., 1997). With anti-inflammatory cytokines or a vasodilator drug (pentoxifyline), this inflammatory pain can be significantly reduced with a decrease in production of cytokines from the resident macrophages (Vale et al., 2003).

Prostaglandins are well-established mediators of inflammation that trigger pain hypersensitivity by promoting nociceptor sensitisation and hyperexcitability. Following activation of the ATP-gated calcium channel P2×4, a multi-step enzymatic cascade that includes the cytosolic phospholipase A2 cyclooxygenase (cPLA2/COX) pathway leads to synthesis of prostaglandin E2 (PGE2), the main prostaglandin produced during the inflammatory response. In resting conditions, tissue-resident macrophages constitutively express P2×4 and stimulation of these receptors in macrophages triggers calcium influx and p38 MAPK phosphorylation, resulting in cPLA2/COX-dependent release of PGE2. However, in response to induced peripheral inflammation, mice lacking the P2×4 receptor do not develop pain hypersensitivity and show a complete absence of inflammatory PGE2 in tissue exudates (Ulmann et al., 2010). The adverse side effects of non-steroidal anti-inflammatory drugs (NSAIDS) calls for the development of new anti-inflammatory drugs with analgesic properties, and so these findings suggest that targeting the macrophage-specific P2×4 receptor could be a useful principle in treating the early stages of osteoarthritis and other inflammatory pain diseases (Jakobsson, 2010). Interestingly, microglia also express the same surface markers as macrophages, including P2×4 receptors, ascribing multiple cellular targets to P2×4 receptor blockade for alleviating inflammatory pain (Zhuo et al., 2011).

Several studies also demonstrate a role of macrophages in neuropathic pain pathology, where a reduction in neuropathic pain behaviours correlates with an attenuation of macrophage recruitment into the damaged nerve (Liu et al., 2000; Myers et al., 1996; Ransohoff, 1997; Sommer and Schafers, 1998). Indeed mice with a delayed Wallerian degeneration show markedly reduced thermal hyperalgesia compared to normal mice with a chronic constriction injury of the sciatic nerve, temporally related to the delayed recruitment of macrophages to the injured nerve (Sommer and Schafers, 1998). Following nerve damage, resident macrophages respond rapidly without the need for prior activation of precursor cells and are joined by circulating macrophages, a process that can occur for 2–3 days after damage. Recruited macrophages quickly outnumber the resident cells and this process is vital for nerve regeneration (Griffin et al., 1993; Taskinen and Roytta, 1997). Their involvement in inflammatory and neuropathic pain makes macrophages an obvious target for study in chronic pain mechanisms but targeting of these cells need to be tempered by the fact that they have a key role in repair.

### 4.4 T-Cells

Lymphocytes are a large group of circulating leukocytes comprising B-lymphocytes, T-lymphocytes and natural killer cells. T-lymphocytes (T-cells) play a central role in cell-mediated immunity by release of cytokines to activate immune cells or through the destruction of infected cells. T-cells are classified either as helper cells (CD4+) or cytotoxic cells (CD8+) with type 1 and 2 subtypes. Th1-cells are responsible for the release of proinflammatory cytokines, whereas Th2-cells release anti-inflammatory cytokines that activate humoral immunity and strongly deactivate macrophages. During an immune response naive T-cells produce Interleukin 2, proliferate and release an array of pro-inflammatory cytokines depending on their subtype (Th1 produce Interferon γ; Th2 produce IL-4, IL-5 and IL-13) (O'Garra and Arai, 2000).

The elimination of subgroups of these cells in animals confirms their central role in acquired immunity and autoimmune diseases. In rheumatoid arthritis, CD4+ T-cells infiltrate the degenerating rheumatoid synovium and produce cytokines—the T-cell blocker abatacept partially inhibits inflammatory disease progression among rheumatoid arthritis patients (von et al., 2012). In rat Freund's adjuvant arthritis, nitric oxide-naproxen has been shown in reduces T-cell proliferation and thereby oedema and pain-related behaviour (Cicala et al., 2000). The infiltration of T-cells into the dorsal horn has also been shown to contribute to the development of neuropathic pain (Costigan et al., 2009).

### 4.5 Glial cells

Microglia, oligodendrocytes and astrocytes constitute the glial cells of the central nervous system. Glial signaling is now understood to be crucial for the development and maintenance of chronic pain (Colburn et al., 1997). Under the normal influence of the CNS microenvironment, microglia exhibit a “surveillance state” with fine, long processes that continually survey their environment. Following activation by pathological events or microbial invasion, the cell morphology, gene expression profile and functional behavior of these cells rapidly changes to the “effector state” resulting in the release of numerous chemokines and cytokines that facilitate an innate immune response (Gao et al., 2009; Zhuo et al., 2011).

**Fig. 2:**
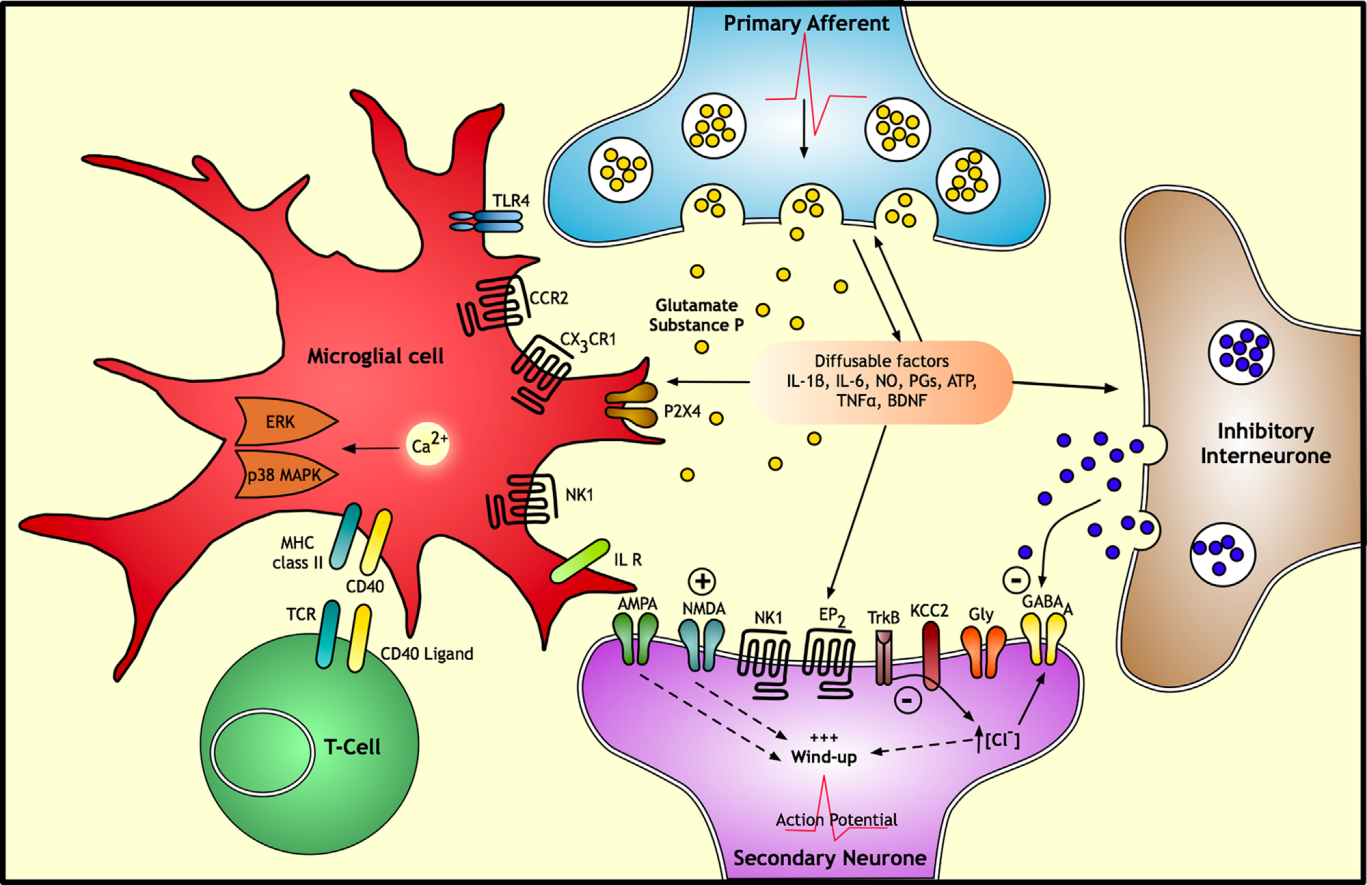
Neuro-immune interactions in the dorsal horn The synaptic transmission between sensory neuronal terminals and dorsal horn neurones is enhanced by activated microglia, T-cells and astrocytes, i.e. after nerve injury. The release of transmitters or modulators from primary afferents stimulates the proliferation and chemotaxis of microglia in the dorsal horn projection region of primary afferents conveying injury. Microglia express MHC-II protein that presents antigens to T-cells that are recruited to the dorsal horn through the aid of co-stimulatory molecules such as CD40 and transient opening of the blood–spinal cord barrier. After initial microglial proliferation (requires the activation of ERK1/2 and p38MAPK), astrocytes proliferate and their processes hypertrophy into an effector state (requires ERK1/2 and JNK). Microglia and astrocytes release several pro-inflammatory cytokines, chemokines and other pro-inflammatory mediators that modulate pain processing by affecting either presynaptic release of neurotransmitters and/or postsynaptic excitability, including as TNFα, IL-1β, IL-6, NO, ATP and prostaglandins. BDNF is also released by microglia that reduces expression of the potassium-chloride exporter KCC2, thereby shifting the anion gradient to allot GABA an excitatory action. All these factors contribute to enhanced excitability of the spinal cord. TNFα: tumour necrosis factor-α; IL-1β: interleukin-1β; IL-6: interleukin-6; NO: nitric oxide; PGs: prostaglandins; BDNF: brain-derived neurotrophic factor; AMPA: α-amino-3-hydroxy-5-methyl-4-isoxazole propionic acid; NMDA, *N*-methyl-d-aspartate; CCR2, CCL2 receptor; CX_3_CR1, fractalkine receptor; MHC-II, major histocompatibility complex type 2; NGF, nerve growth factor; NK1R, neurokinin-1 receptor; P2X4, ionotropic purinoceptors; TLR4, Toll-like receptor 4; ERK, extracellular signal-regulated kinase; p38 MAPK, p38 mitogen-activated protein kinase.

Glial cells are activated by several neuronal-derived signals and thus express an array of receptors (Fig. 2), i.e. microglia have P2X and P2Y receptors for ATP, CX_3_CR1 for fractalkine, neurokinin-1 for substance P and CCR2 for monocyte chemotactic protein (MCP-1) and erbB2, 3 and 4 for neuregulin-1. Activated microglia release several pro-inflammatory cytokines, chemokines and growth factors such as brain-derived neurotrophic factor (BDNF) that modulate nociceptive processing by altering presynaptic release of neurotransmitters and/or postsynaptic excitability. Inflammatory mediators released include TNFα, IL-1β, IL-6, nitric oxide (NO), ATP and prostaglandins (PGs), which initiate a selfpropagating mechanism of further cytokine expression, ultimately leading to an increase in intracellular calcium and activation of the downstream p38 and MAPK/ERK pathway within microglia (Zhuang et al., 2005).

Both microglia and astrocytes are activated in response to nerve injury, The chemokine CCL2 is produced and released in an activity-dependent manner by damaged and undamaged primary afferents in neuropathic rats, and intraspinal administration of CCL2 in naïve rats can activate spinal microglia to produce neuropathic pain-like behaviour (Thacker et al., 2009). CCR2 knockout mice also fail to develop tactile allodynia following nerve injury (Abbadie et al., 2003). Fractalkine is constitutively expressed by neurones of the spinal cord and dorsal root ganglia, and its receptor CX_3_CR1 in microglia is upregulated in a regionally specific manner in two neuropathic pain models (Verge et al., 2004). In the context of nerve injury microglia secrete cathepsin S that cleaves transmembrane fraktalkine, which upon release binds to its receptor on microglia promoting a ‘pro-algesic’ phenotype (Clark et al., 2007). Furthermore, neutralising antibody against rat CX_3_CR1 delays the development of mechanical allodynia and thermal hyperalgesia, suggesting that prolonged release of fractalkine may contribute to the maintenance of neuropathic pain (Milligan et al., 2004). Neuregulin-1 (Nrg1) is expressed by DRG cells and released upon peripheral nerve injury binding to erbB tyrosine kinase receptors on microglia. In vitro Nrg1 promotes microglial survival, proliferation, chemotaxis and Il-1 release and in vivo contributes to the development of microgliosis and neuropathic pain (Calvo et al., 2010).

Intraplantar and sciatic nerve injection of a more novel pro-inflammatory cytokine, IL-17, induces mechanical allodynia and thermal hyperalgesia associated with increased neutrophil infiltration in mice. IL-17 knockout mice also show attenuated mechanical pain hypersensitivity and decreased infiltration of T-cells and macrophages to the injured sciatic nerve, associated dorsal root ganglia and spinal cord segments in neuropathic mice (Kim and Moalem-Taylor, 2011). IL-17 thus contributes to the regulation of immune cell infiltration and glial activation following peripheral nerve injury.

Given the increasing evidence that glia play key roles in neuropathic pain, these cells and their signaling molecules are promising pharmacological targets for analgesic therapies. Even the pharmacological inhibition of microglial activation, i.e. using the second-generation tetracycline minocycline, can attenuate behavioural hypersensitivities exhibited in neuropathy (Mika et al., 2009). The therapeutic benefits of targeting glial signaling molecules include fewer side effects on acute pain sensations given that these molecules are predominantly upregulated only in activated microglia, and thus only in pathological states (Zhuo et al., 2011). However, this promise has not yet translated into clinical benefit (Landry et al., 2012).

### 4.6 Discussion

Here we have highlighted the role of some immune cells in nociceptive signaling and the generation of chronic pain states. Neuro-immiune interactions are now thought to be essential in both the peripheral and central nervous system, notably exemplified by critical roles of the pro-inflammatory cytokines IL-1β and TNF-α in maintaining pain behaviours. Indeed we cannot disregard the potential role of the ‘non-immune’ Schwann cells in nociceptive circuits, given their intimate contact with all sensory neurones and their synthesis of pro-inflammatory mediators (e.g. NGF, TNF-α, IL-1β, IL-6) that amplify the pool of signaling molecules also released by immune cells.

## 5 Cancer-induced bone pain

Treating pain related to cancer is a clinical challenge in itself, and a common problem is cancer-induced bone pain (CIBP) (Mercadante, 1997). Despite drawing parallels with other pain conditions, CIBP is a unique condition encompassing features of both neuropathic and inflammatory pain by producing numerous changes along the neuraxis and remains a considerable therapeutic challenge in the clinical setting (Laird et al., 2010).

### 5.1 Clinical features

CIBP manifestations are multifaceted comprising of tonic pain (ongoing), incident pain (pain on movement) and break-through pain (manifests at rest or on movement being both intense and unpredictable) (Laird et al., 2010). These different components can occur as isolated events or in tandem and effective pain relief in CIBP may require individual attention to each aspect. The first symptom of metastatic bone cancer is usually the presence of a constant dull and throbbing pain, the quality of which intensifies as the disease evolves in response to progressive destruction of bone (Mercadante, 1997). As the disease advances, episodes of incident pain and break-through pain occur more frequently and with greater intensity (Mercadante, 1997; Portenoy and Lesage, 1999). In the majority of these patients, opioid analgesia is the preferred and sometimes only method of providing pain relief. However, treatment for these painful episodes is hampered by the need of higher than normal doses of opioids, which are accompanied by side effects such as sedation, constipation, tolerance and opioid-induced hyperalgesia (Portenoy and Hagen, 1990; Portenoy and Lesage, 1999; Serafini, 2001).

Break-through pain occurs in more than 50% of cancer patients and is predictive of more severe pain, emotional distress, physical disabilities and poor quality of life (Caraceni and Portenoy, 1999; Portenoy and Lesage, 1999). A recent study profiling the characteristics of CIBP correlated the worst pain experienced by patients with break-through pain and the associated functional impairment (Laird et al., 2010). Although break-through pain occurs transiently, it is reported to have detrimental socio-economic impacts due to work loss and hospitalisation-related medical costs (Fortner et al., 2002).

### 5.2 Current CIBP therapies

Management of pain relief is key in maintaining quality of life in patients with metastatic bone disease (Vasudev and Brown; 2010). Treatment for CIBP often comprises pharmacological and non-pharmacological approaches, including administration of various analgesics, bisphosphonates, radiation and surgery (Levy, 1996; Vasudev and Brown; 2010).

**Fig. 3:**
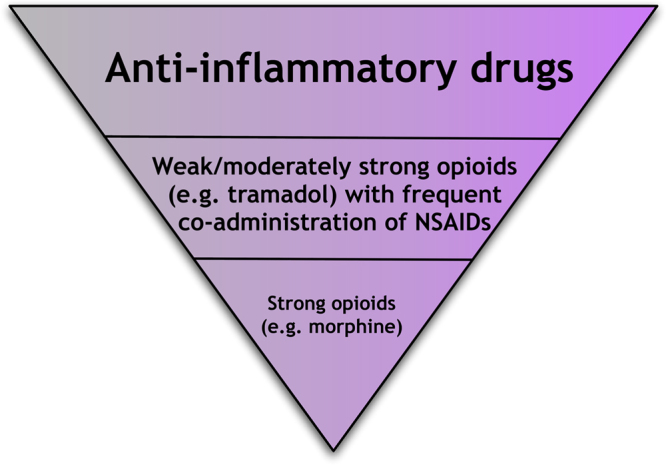
World Health Organisation (WHO) treatment algorithm. Benefits from using NSAIDs in the management of cancer pain include low cost and wide availability, familiarity to patients and ease of administration (step 1). Combinations of NSAIDS and opioids (steps 2 and 3) are recommended for moderate to severe cancer pain.

Because cancer pain is a complicated mixture of nociceptive, inflammatory, visceral and neuropathic pains, its treatment requires a multi-modal therapeutic approach. NSAIDs are advocated for use in mild to moderate cancer pain on steps 1 and 2 of the World Health Organisation treatment algorithm for cancer pain (Fig. 3).

Opioids can be used as stand-alone medications or in combination with NSAIDs to treat around-the-clock cancer pain. Although opioids are chosen for individual patients on the basis of tolerability and accounting for the risk to benefit ratio for pain management, their pharmacokinetic and pharmacodynamic profile is given precedence for treating break-through pain (Colvin and Fallon, 2008).

Oral transmuscosal fentanyl citrate is reported to produce lower pain intensity and higher pain relief scores among patients with a positive response to opioid treatment (Coluzzi et al., 2001). It is the only medication currently licensed for management of breakthrough pain in patients who are on maintenance opioid therapy for underlying chronic cancer pain. Like most opioids, transmuscosal fentanyl citrate requires dose titration to find the minimal effective dose and product information in the UK suggests that no more than 4 units of the minimal effective dose should be administered per day. This suggests a potential limitation in the number of rescue medication doses administered to patients who experience more than four daily painful episodes. Accordingly, up-titration and dose adjustments to find a new minimal effective dose may delay pain relief.

A recent multi-center European study investigating break-through cancer pain found that only 52 of 320 patients studied experienced complete relief with their underlying and rescue medication (Davies et al., 2011). This is unsurprising given that the majority of patients were receiving a modal dose of either oral morphine or oxycodone as both underlying and rescue medication, and the pronlonged onset and peak effect for such preparations may be inadequate to deal with the break-through pain associated with metastatic bone pain. A study of CIBP patients has reported that breakthrough pain has a very rapid onset (<5 min) and a short duration (15 min) compared to other cancer pains (Laird et al., 2010).

**Fig. 4:**
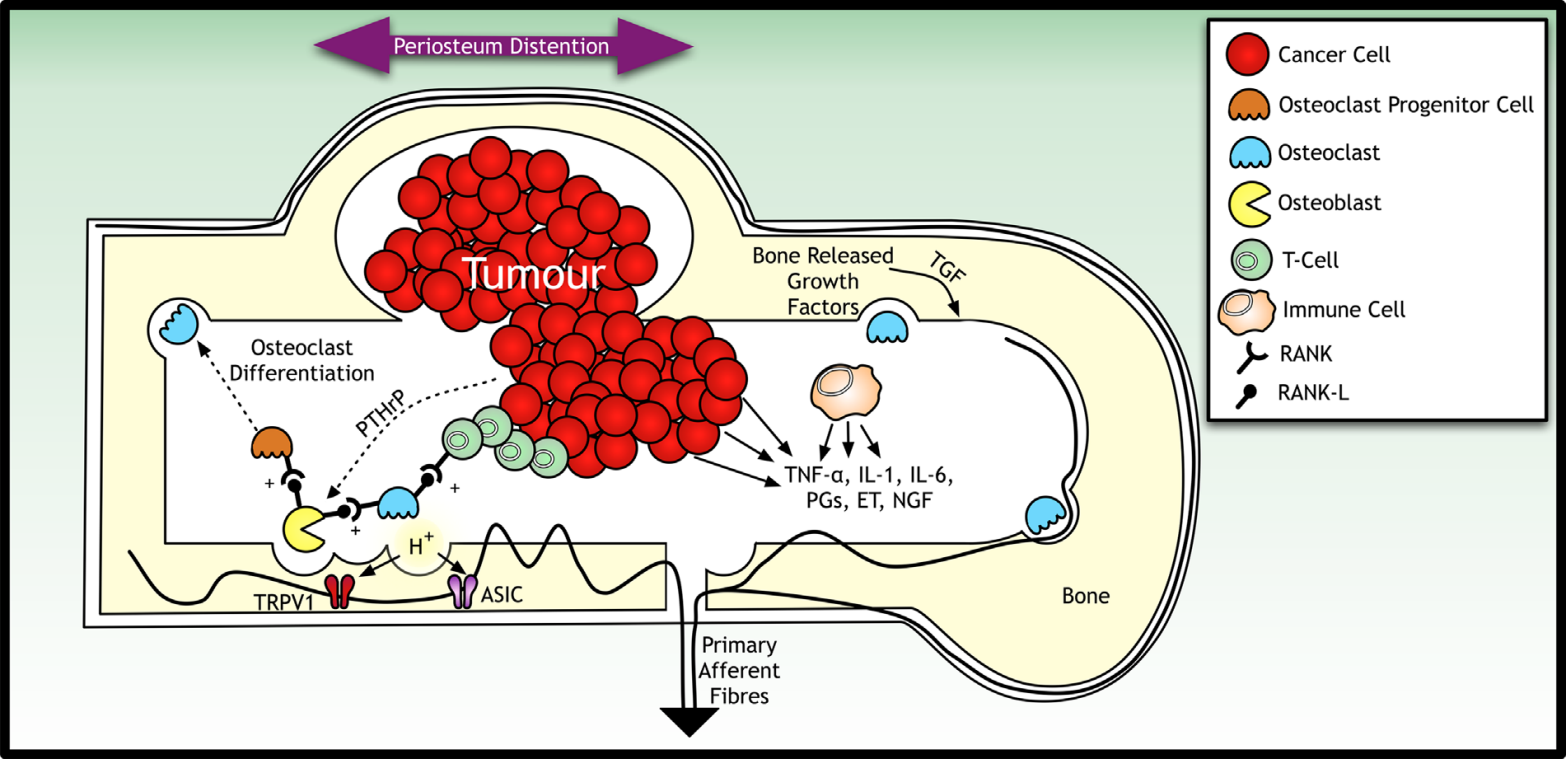
Factors leading to peripheral sensitisation in osteolytic tumours. Under normal conditons, osteoblast and osteoclast activity is coupled to permit appropriate remodeling of the bone. Osteoblasts express RANK-L, a member of the tumour necrosis family that binds to RANK, which is found on osteoclasts and osteoclast progenitor cells to increase their activation and differentiation, respectively. In the presence of a tumour, immune cells are recruited to secrete various pro-inflammatory mediators in response to a change in the microenvironemnt. T-cells are can stimulate bone resorption independent of osteoblast activity (due to their expression of RANK-L). Osteoclast activity is dependent on a low pH environment, and the activation of ASIC and TRPV1 channels by protons derived from osteoclast activity can facilitate nociceptive processing. Moreover, structural weakening of the bone induced by increased osteoclast activity may distend the highly innervated periosteum to amplify the afferent barrage into the central nervous system and drive pain perception. RANK receptor activator of nuclear factor κB-ligand; RANK-L: RANK ligand; TNFα: tumour necrosis factor-α; IL-1/6: interleukin-1/6: NGF: nerve growth factor; PGs: prostaglandins; ET: endothelin.

Bisphosphonates bind to areas of active bone metabolism to inhibit osteoclastic bone resorption (Fig. 4) and thereby decrease the osteolytic effects of a tumour. The most commonly prescribed bisphosphonates for the treatment of bone metastases are clodronate, pamidronate and zoledronic acid, although only the more potent nitrogen containing bisphophonates; pamidronate and zoledronic acid are approved for the treatment of metastatic cancer in the US (Coluzzi et al., 2011).

Three large-scale clinical trials investigating the use of bisphosphonates in metastatic bone disease identified key biological biomarkers, including bone-specific alkaline phosphotase and skeletal-related events (Major and Cook, 2002). Skeletal-related events were defined as: pathologic fracture, spinal cord compression, occurrence of bone pain that required palliative radiation therapy, surgery to bone or hypercalcemia of malignancy. The number of and time to first skeletal-related event during the study was the primary efficacy measure employed in all three trials, and as a result became the basis for drug approval for treatment of bone metastases in the US (Coleman et al., 2005; Ibrahim et al., 2003). The synonymous occurrence of a skeletal-related event with the occurrence of pain likely underlies the analgesic effects of bisphosphonates in patients with osteoclast-induced skeletal metastases (Coluzzi et al., 2011). Nevertheless, the analgesic effects produced by bisphosphonates in combination with opioids are thought to be modest (Mercadante, 1997). As with NSAIDs, bisphosphonates are associated with gastrointestinal tract toxicity, fever, and electrolyte abnormalities (Mantyh, 2002).

### 5.3 New targets for CIBP management

Gabapentinoids are not licensed for the management of CIBP, but preclinical data suggests that they may prove an effective treatment for metastatic cancer pain. In a rat model of CIBP, chronic treatment with gabapentin was found to ameliorate pain behaviours (Donovan-Rodriguez et al., 2005). Furthermore it was previously reported by the same group that there is phenotypic shift in lamina I neurones from predominantly NS like to WDR-like neurones (Urch et al., 2005). Chronic treatment with gabapentin was further found to reverse these pathophysiological changes in lamina I back to a desensitised state (Donovan-Rodriguez et al., 2005).

One early proof-of-concept study investigated the cytokine RANK/RANK-L interaction in metastatic cancer using osteoprotegrin, a decoy receptor for RANK-L (receptor activator of nuclear factor κB-ligand). RANKL/RANK signaling regulates the formation of multinucleated osteoclasts and their activation in normal bone remodelling (Body et al., 2003). Osteoprotegrin protects the skeleton from excessive bone resorption by binding to RANK-L and preventing it from binding to its receptor, RANK (Boyce and Xing, 2007). In 2003 a phase I randomised dose escalation study determined both the safety and the effect of AMGN-0007 (a recombinant osteoprotegrin) on bone resorption (Body et al., 2003). Subcutaneous AMGN-0007 significantly suppressed bone resorption compared to intravenous bisphosphonate pamidronate measured by urinary *N*-telopeptide of collagen (NTX) (a surrogate biomarker of bone resorption). Denosumab is a non-cytotoxic IgG2 monoclonocal antibody for the RANK-L ligand expressed on osteoclasts and has been investigated in bisphosphonate naive patients with breast cancer related metastases (Lipton et al., 2007, 2008). Skeletal-related events were reported more frequently and urinary NTX levels higher among intravenously bisphosphonate-treated patients compared to the denosumab group, implicating a similar if not better efficacy profile of denosumab compared to bisphophonates, also in terms of delaying or preventing skeletal- related events (Fizazi et al., 2011; Henry et al., 2011; Stopeck et al., 2010). Together these studies set a strong precedence for the use of denosumab as an alternative therapy in the armoury of medications used to manage metastatic bone pain.

Another potential treatment for CIBP includes anti-nerve growth factor (anti-NGF) antibodies, e.g. tanezumab. The release of NGF by cancer cells (Sevcik et al., 2005) and the NGF-induced sensitisation of primary afferent nerves in the tumour-laden bone (Pantano et al., 2011) highlight potential mechanisms for pain relief with anti-NGF antibodies. A recently completed phase II trial has investigated the safety and efficacy of tanezumab as an add-on therapy to opioids in treating pain related to bone metastases (clinicaltrial.gov NCT00545129). However, the same approach used in patients with osteoarthritic pain of the knee saw the studies halted early due to early joint replacement in patients administered tanezumab compared to those who received placebo (Lane et al., 2010).

A novel molecule currently under clinical investigation by Sanofi Aventis includes SSR411298, a fatty acid amide hydrolase (FAAH) inhibitor under evaluation as an adjunctive treatment for persistent cancer pain for patients receiving WHO Step 2 and 3 cancer pain treatments (clinical trials.gov NCT 01439919). FAAH is one of two principle enzymes responsible for the hydrolysis of the endocannabinoids: *N*-arachidonoyl ethanolamine (anandamide) and 2-arachidonoylglycerol. Both tetrahyrdocannabinol, the psychoactive component of marijuana, and cannabinoid receptor 1 antagonists are known to have analgesic properties, however these are tainted by undesirable side-effects which limit their usefulness (Ahn et al., 2009). The primary objective of the study will evaluate the safety and efficacy of SSR411298 200 mg daily compared to placebo as measured by a change in pain severity from baseline using the numerical rating scale.

More recent approaches seek to improve the efficacy or durability of licensed medications for the treatment of metastatic cancer pain, including fentanyl buccal tablets with oxycodone (Clinicaltrials.gov NCT00463047). Fentanyl is a fast acting opioid generally used for the management of cancer break-through pain, given its faster onset of action. Similarly, ketamine a *N*-methyl-d-aspartate (NMDA) receptor antagonist may improve analgesia in patients with uncontrolled pain receiving high doses of opioids and may also prove an effective adjuvant that reduces opioid consumption and tolerance in patients with CIBP (clinicaltrials.gov NCT00484484).

### 5.4 Summary

A number of new targets are arising and are under review for the management of CIBP. Numerous *in vivo* preclinical studies and human studies, some of which we have discussed here, have demonstrated that metastatic cancer pain is distinct from other chronic pain states, yielding a need to improve currently prescribed therapies and produce novel approaches to tackle this debilitating yet poorly understood condition.

## 6 Summary

We have discussed the science and clinical picture of four topics in pain research that have received growing interest in recent literature. For some heritable pain states, the contributing genetic factors have been identified with increasing sophistication of genetic profiling techniques. Further preclinical modeling may be able to bridge the knowledge gap between the effects of altered nociceptor excitability at the molecular level to the underlying pathophysiology. We have also reviewed mediators of cold sensory processing as well as non-neuronal cells in the inflammatory response. Indeed, a body of literature has now identified the recruitment of specific immune cells and release of particular pro-inflammatory mediators following insult that contribute to chronic or persistent pain. Our last topic of review was the challenges in treating cancer-induced bone pain and the need to improve current therapies for this unique condition. Overall, we have provided an overview of both the molecular and cellular mechanisms and also the clinical manifestations of the reviewed topics. Linking the ties between preclinical and clinical data will yield further key insights into the mechanistic understanding of pain pathophysiology and hopefully translate into effective analgesic treatments.

## Figures and Tables

**Table 1:** Inherited pain syndromes and associated channel dysfunctions. PE: Primary erythromelalgia; PEPD: paroxysmal extreme pain disorder; CIP: Na_v_1.7-associated congenital insensitivity to pain; FHM: familial hemiplegic migraine; HSAN: hereditary sensory and autonomic neuropathy; FEPS: familial episodic pain syndrome.

Inherited disorder	Gene (protein)	Gain (+)/loss (+)	Change in channel function	Pathophysiology
PE	*SCN*9*A* (Na_V_1.7)	+	Hypolarized voltage-dependence (reduced activation theshold) and slowed deactivation	Nociceptor hyperexcitability
PEDP	*SCN*9*A* (Na_V_1.7)	+	Impaired Inactivation	Nociceptor hyperexcitability; persistent sodium currents/repetitive neuronal firing
CIP	*SCN*9*A* (Na_V_1.7)	−	Frameshift splicing alteration and premature mtermination of protein	Impaired nociceptor function
FHM1	*CACNL*1*A*4 (Ca_v_2.1)	+	Reduced activation threshold and enhanced open channel probability	Enhanced cortical spreading depression
FHM2	*ATP*1*A*2 Na^+^/K^+^ ATPase	−	Impaired pump action	Increased K^+^ in extracellular space
FHM3	*SCN*9*A* (Na_V_1.1)	+/−	Loss or gain of function depending on mutation type	Neuronal hyperexcitability
HSAN-V	*NGF* (β*-*NGF)	−	Impaired β-NGF signaling through p75^NTR^	Reduced nociceptive acitivity
FEPS	*TRPA* (TRPA1)	+	Increased activation current at resting membrance prtential	Excessive neuronal firing
